# Effectiveness of fast-track pathway for diabetic foot ulcerations

**DOI:** 10.1007/s00592-021-01721-x

**Published:** 2021-05-03

**Authors:** Marco Meloni, Jose Luis Lazaro-Martínez, Raju Ahluwalia, Benjamin Bouillet, Valentina Izzo, Michela Di Venanzio, Elisabetta Iacopi, Chris Manu, José Luis Garcia-Klepzig, Juan Pedro Sánchez-Ríos, Claas Lüedemann, Víctor Rodriguez-Saenz De Buruaga, Julien Vouillarmet, Jérôme Guillaumat, Anna Rita Aleandri, Laura Giurato, Micheal Edmonds, Alberto Piaggesi, Kristien Van Acker, Luigi Uccioli

**Affiliations:** 1 Diabetic Foot Unit, University of Rome Tor Vergata, Viale Oxford 81, 00133 Rome, Italy; 2 Diabetic Foot Unit, Universidad Complutense de Madrid, Madrid, Spain; 3 Department of Trauma and Ortophaedic Department, King ´s College Hospital, London, UK; 4 Endocrinology Department, University Hospital Center, Dijon, France; 5 UOSD Diabetologia San Camillo De Lellis Hospital, Rieti, Italy; 6 Diabetic Foot Section, University of Pisa, Ospedale Di Cisanello, Pisa, Italy; 7 Diabetic Foot Clinic, King’s College Hospital, Denmark Hill, London, UK; 8 Internal Medicine Department, Hospital Clinico San Carlos De Madrid, Madrid, Spain; 9 Diabetic Foot Unit, Vascular Surgery Department, Hospital Fundación Alcorcón, Madrid, Spain; 10 Franziskus Krankenhaus, Berlin, Germany; 11 Vascular Surgery Department, Donostia Hospital Universitario San Sebastian, San Sebastian, Spain; 12 Endocrinology Department, University Hospital Center, Lyon, France; 13 University Hospital of Caen, Caen, France; 14 Department of Endocrinology, Familie Ziekenhuis, Chimay, Belgium

**Keywords:** Diabetes, Diabetic foot, Early referral, Healing, Limb salvage, Wound care

## Abstract

**Aim:**

To investigate the effectiveness of fast-track pathway (FTP) in the management of diabetic foot ulceration (DFU) after 2 years of implementation.

**Methods:**

The study group was composed of patients who referred to a specialized DF centre due to DFUs. Those were divided in two groups: early referral (ER) and late referral (LR) patients. According to FTP, ER were considered patients who referred after 2 weeks in the case of uncomplicated non-healing ulcers (superficial, not infected, not ischemic), within 4 days in the case of complicated ulcers (ischemic, deep, mild infection) and within 24 h in the case of severely complicated ulcers (abscess, wet gangrene, fever). Healing, healing time, minor and major amputation, hospitalization, and survival were evaluated. The follow-up was 6 months.

**Results:**

Two hundred patients were recruited. The mean age was 70 ± 13 years, 62.5% were male, 91% were affected by type 2 diabetes with a mean duration of 18 ± 11 years. Within the group, 79.5% had ER while 20.5% had LR. ER patients showed increased rates of healing (89.9 vs. 41.5%, *p* = 0.001), reduced healing time (10 vs. 16 weeks, *p* = 0.0002), lower rates of minor (17.6 vs. 75.6%, *p* < 0.0001) and major amputation (0.6 vs. 36.6%, *p* < 0.0001), hospitalization (47.1 vs. 82.9%, *p* = 0.001), and mortality (4.4 vs. 19.5%, *p* = 0.02) in comparison to LR. At multivariate analysis, ER was an independent predictor of healing, while LR was an independent predictor for minor and major amputation and hospitalization.

**Conclusion:**

After the FTP implementation, less cases of LR were reported in comparison to ER. ER was an independent predictor of positive outcomes such as healing, healing time, limb salvage, hospitalization, and survival.

## Introduction

Delayed referral of persons with diabetic foot ulceration (DFU) to specialised diabetic foot services (DFS) is still a common concern around the world. It has been reported that approximately in 60% of cases, the duration of DFU was unknown or DFU diagnosis was delayed more than three weeks from the onset of the wound [1]. In addition, health care providers working in primary care, mainly general practitioners (GPs), reported that in 40% of case they are not adequately trained in the management of DFUs [2].

Due to this current issue, International Diabetic Foot Care Group (IDFCG) and D-Foot International developed an easy-to-use tool, called fast-track pathway (FTP) for DFU, addressed to not-expert health care professionals (HCPs) and aiming to detect ulcer’ severity, the specific management and timing of referral [3]. The aim of the FTP was to promote early referral of persons with DFUs to specialised DFS and improving healing, avoiding lower-limb amputations, and reducing mortality.

Since its inception and publication, the FTP has been distributed by IDFCG and D-Foot International through International [3]. It has gained national and regional recognition and was presented at local meetings to HCPs working in primary care such as GPs, district nurses, and in other settings such as diabetologists, podiatrist, vascular & orthopaedic surgeons, interventional radiologists/cardiologists among others.

From 2018, the FTP [3] has been adopted in the Italian Health Care community by the support of Italian Diabetic Foot Study Group and expert key leaders in the management of diabetic foot disease. It was advocated by several professional involved in the DF care including primary care and specialized DFS. Accordingly, the FTP was also implemented in the region of Rome and Lazio which feed to a tertiary level multidisciplinary diabetic foot team (MDFT) specialist clinic serving a large diabetic foot population.

This study aims to evaluate the efficacy of FTP implementation at a regional level, in a developed country with what is considered to be a mature DFS, and its effect on patients presenting with Diabetic Foot Disease and their primary outcomes.

## Methods

### Patient selection

**Fig. 1:**
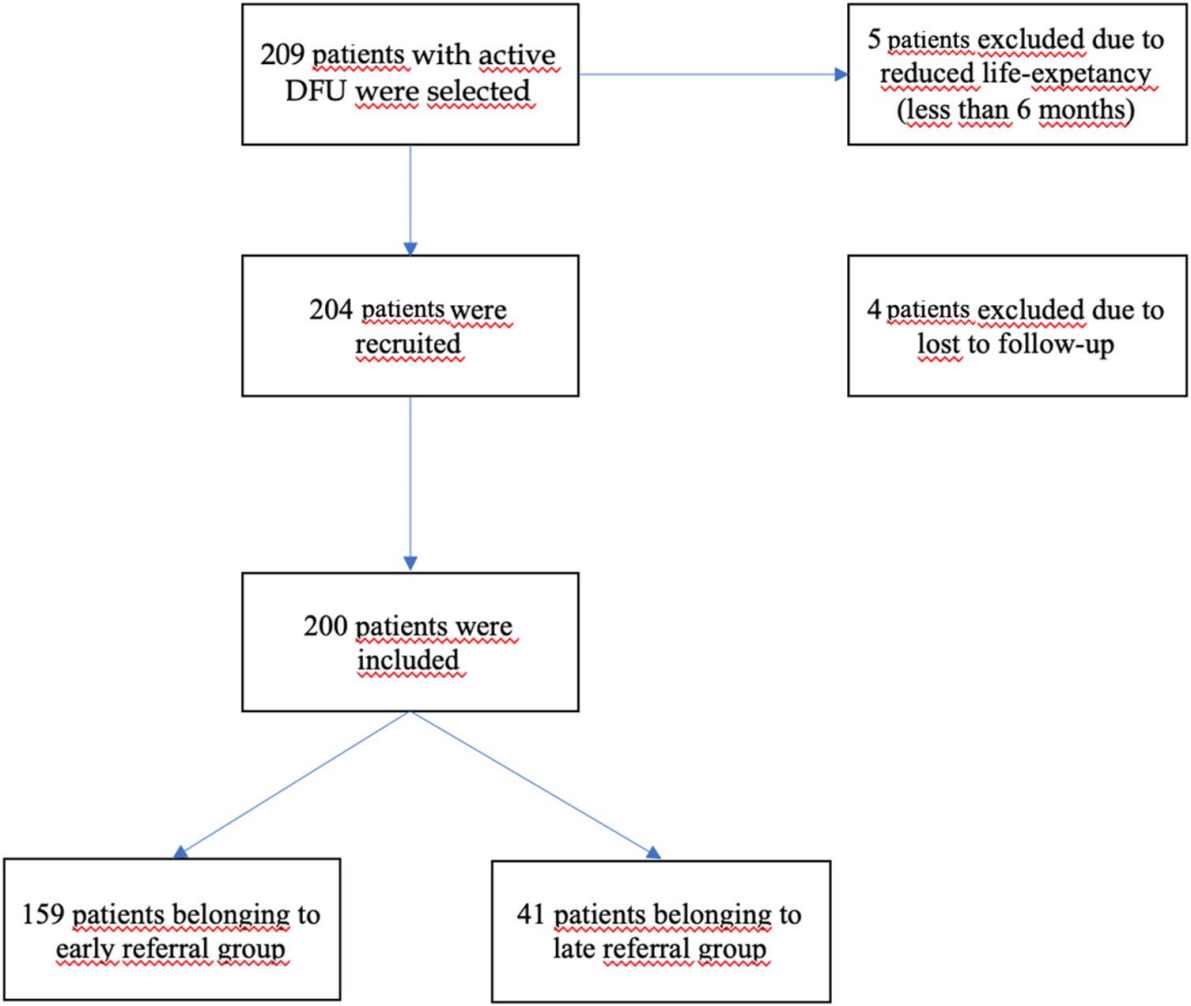
Flow-chart on patients’ recruitment

This study is a retrospective study conducted in a single centre. Consecutive patients who referred to a tertiary level DFS serving Rome and Lazio, Italy since January 2019–May 2020 for a new active DFU were included in the study while patients with reduced life-expectancy (less than 6 months) were excluded (see Fig. 1).

All patients were managed by a pre-set limb salvage protocol following International Working Group on the Diabetic Foot (IWGDF) Guidance [4], including restoration of foot perfusion in the case of peripheral ischemia, antibiotic therapy (and surgery if required) in the case of infection, offloading of affected limb, management of diabetes and comorbidities, ulcer debridement, and local wound care based on the best evidence recommendations.

At the time of admission, demographic and clinical data as well as DFU characteristics were recorded. In addition, DFU’ severity and timing of referral were reported according to the FTP classification and recommendations.

### Clinical assessment

Hypertension was considered in the case of current antihypertensive therapy; hypercholesterolemia was considered in the case of current statin therapy; ischaemic heart disease (IHD) was considered in the case of previous acute coronary syndrome or coronary revascularization, evidence of angina, significant changes on electrocardiography (above or under-levelling ST, *q* wave, inversion of T wave, new left bundle branch block). Cerebrovascular disease (CVD) was considered in the case of previous cerebrovascular ischemia, previous carotid revascularization, or significant carotid artery disease (occlusion > 70%). Dialysis was considered in the case of end-stage-renal-disease (ESRD) requiring renal replacement therapy.

### Ulcer characteristics

Ulcer characteristics reported at the time of presentation, and first assessment at the MDFT. Deep ulcers were considered in the case of full thickness skin lesions, extending from the subcutaneous to tendon, muscle, or bone. Diagnosis of infection was defined according to IWGDF guidelines [4].

Standard treatment in the centre followed IWGDF guidelines with initial broad spectrum antibiotic therapy and then according on culture results if required [4]. All patients received off-loading for relieving pressure and trauma in the ulcer area according both to ulcer location, the presence of both ischemia or infection (isolated or in conjunction) and individual needs [4].

The association of ischaemic, and ulceration was defined as either no palpable distal pedal pulses, TcPO2 < 30mmhg [4, 5] and/or arterial stenosis/occlusions documented by ultra-sound duplex or computed tomography or MRI requiring lower limb revascularization. The revascularization procedure was performed in respect to foot condition, vessels affected and patient’s general condition by either endovascular or surgical (by-pass) procedure [4, 6].

### Assessment of DFU severity

DFU’ severity and timing of referral were classified according to FTP classification [3]: uncomplicated DFUs were considered in the case of superficial, not infected, not ischaemic ulcers; complicated DFUs were considered in the case of ischaemic, infected (mild/moderate), deep (involving soft tissue and/or bone), or any kind of ulcers in patients on dialysis or with heart failure; severely complicated DFUs were considered in the case of abscess, wet gangrene, necrotizing fasciitis or in the case of fever or clinical signs of sepsis.

### Assessment of referral timing

According to the timing of referral, patients were divided in two groups: early referral (ER) and late referral (LR). Based on the FTP recommendations [3], ER were considered patients who referred immediately after 2 weeks in the case of uncomplicated non-healing ulcers (reduction of ulcer size < 30% after 2 week of standard of care), within 4 days in the case of complicated ulcers and within 24 h in the case of severely complicated ulcers. LR patients were considered when the specific timing of referral for each grade of ulcer’ severity was not respected.

### Clinical outcomes

Completed ulcer healing, healing time, minor and major amputation, hospitalization, and survival after at least 6 months of follow-up were evaluated. Definitive ulcer healing was taken to be complete epithelialization of the target wound, and maintenance of the closed healed epithelized surface for a minimum of 2 weeks. Healing time was reported in weeks. Minor amputation was considered any amputation below-the-ankle (digital, ray, metatarsal, Lisfranc, Chopart). While, major amputation was considered any amputation above the ankle. Mortality for any cause was recorded.

### Statistical analysis

Data are expressed as mean ± SD. Comparison between groups was reported using an *X*^2^ test (frequency data) or Student’s *t* test (continuous data). Univariable logistic regression analyses was performed for all potential predictor variables with the outcome of interest (major amputation and mortality), with values presented as univariable odds ratios (ORs) along with the respective 95% CI. Thereafter, all potential predictors were entered simultaneously into a multivariable logistic regression model. These models yielded a set of variables that best predict outcome. Statistical analysis was performed by SAS (JMP12; SAS Institute, Cary, NC) for the personal computer.

## Results

Two hundred four patients were recruited. Four patients were excluded due to loss to follow-up. Within the group, 159 (79.5%) had ER while 41 (20.5%) had LR (see Fig. 1).

### Demographics and patient ulcer characteristics related to referral times

The mean age was 70 ± 13 years, 125 (62.5%) were male, 182 (91%) were affected by type 2 diabetes with a mean duration of 18 ± 11 years (see Table 1). Twenty-one patients (10.5%) showed uncomplicated DFUs, 123 (61.5%) complicated DFUs and 56 (28%) severely complicated DFUs. One-hundred eleven patients (55.5%) were ischaemic, 135 (67.5%) were infected, and 119 (61.5%) showed deep to the bone DFUs. In addition, 127 (63.5%) patients had DFUs larger than 5 cm^2^ and 85 (42.5%) had a gangrene (see Table 1). ER subjects were older and had a lower duration of diabetes, however more cases of ESRD and IHD (see Table 1).

**Table 1:** Baseline clinical and diabetic foot ulcers’ characteristics of all sample, early reffereral and late referral groups

Variables	All sample (*n* = 200)	Early referral (*n* = 159)	Late referral (*n* = 41)	*P*
Age (years)	70 ± 13	71 ± 13	66 ± 13	0.03
Sex (male) (*n*) (%)	125 (62.5)	98 (61.6)	27 (63.4)	0.7
Diabetes (type 2) (*n*) (%)	182 (91)	149 (93.7)	33 (80.5)	0.04
Diabetes duration (years)	18 ± 11	17 ± 8	23 ± 17	0.003
HbA1c (mmol/mol) (%)	60 ± 10 (7.6 ± 3.3)	58 ± 9 (7.5 ± 3.2)	62 ± 18 (7.8 ± 3.8)	0.08
ESRD (*n*) (%)	25 (12.5)	14 (8.8)	11 (26.8%)	0.004
IHD (*n*) (%)	78 (39)	58 (36.5)	20 (48.8)	0.1
Hypertension (*n*) (%)	193 (96.5)	154 (96.9)	39 (95.1)	0.6
Dyslipidemia (*n*) (%)	158 (79)	120 (70.5)	38 (92.7)	0.008
COPD (*n*) (%)	26 (13)	19 (11.9)	7 (17.1)	0.4
CVD (*n*) (%)	36 (18)	28 (17.6)	8 (19.5%)	0.7
*Ulcer severity*				
Uncomplicated DFU	(10.5)	21 (13.2)	0 (0)	< 0.0001
Complicated DFU	(61.5)	106 (66.7)	17 (41.5)	
Severely complicated	(28)	32 (20.1)	24 (58.5)	
Ischaemia	111 (55.5)	80 (50.3)	31 (75.6)	0.002
Infection	135 (67.5)	103 (64.8)	32 (78)	0.07
Deep to the bone	119 (61)	88 (55.3)	31 (75.6)	0.009
Dimension (> 5 cm^2^)	127 (63.5)	91 (57.2)	36 (90)	0.003
Gangrene	85 (42.5)	52 (37.7)	33 (80.5)	< 0.0001

*HbA1c* hemoglobin A1c; *ESRD* end-stage-renal-disease; *IHD* ischaemic heart disease; *COPD* chronic obstructive pulmonary disease; *CVD* cerebrovascular disease; *DFU* diabetic foot ulcer

### Prognostic factors for ulcer outcomes

**Fig. 2:**
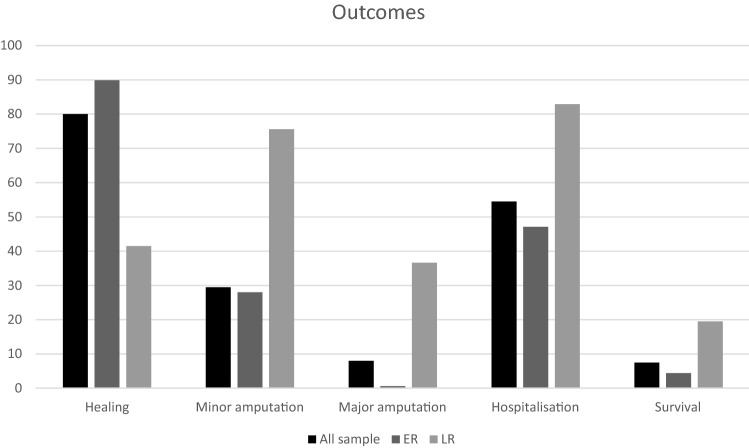
Outcome of, respectively, all samples, early referral and late referral groups: healing (80, 89.9 and 41.5%, *p* = 0.0001), minor amputation (29.5, 28, 79.5%, *p* < 0.0001), major amputation (8, 0.6, 36.6%, *p* = 0.0001), mortality (7.5, 4.4, 19.5%, *p* = 0.02). *ER* early referral; *LR* late referral

We observed lower rates of severely complicated, ischeamic, infected, large (> 5 cm^2^) DFUs and gangrene in the ER group, and increased rates of healing, reduced healing time, lower rates of minor and major amputation, hospitalizations, and mortality (Fig. 2). Further, multivariate analysis of all predictors identified at univariate analysis indicated ER and absence of ESRD requiring dialysis were independent predictors of healing. While LR and gangrene were independent predictors for minor amputation, LR was an independent predictor of major amputation. The combination of LR and ischemia was independent predictors of hospitalization, and IHD was independent predictor of death (see Tables 2 and 3).

**Table 2:** Multivariate analysis of independent predictors of outcome (healing, minor amputation, major amputation)

Variables	Healing			Minor amputation			Major amputation		
	OR	95%	*P* value	OR	95%	*P* value	OR	95%	*P* value
Absence of ESRD	1.7	1.2–2.5	0.02				1.3	0.5–1.5	0.8
Infection				0.8	0.6–1.3	0.5			
Early referral	8.5	3.8–15.4	0.0001						
Late referral				7.3	2.5–18.5	0.0001	4.9	1.9–10.6	0.0002
Ischemia	0.9	0.6–1.5	0.4						
Gangrene	1.1	0.3–1.4	0.3	2.9	1.7–4.2	0.006			
IHD	1.2 0	8–1.7	0.06						

*ESRD* end-stage-renal-disease; *IHD* ischaemic heart disease

**Table 3:** Multivariate analysis of independent predictors of outcome (hospitalization, mortality)

Variables	Hospitalization			Mortality		
	OR	95%	*P* value	OR	95%	*P* value
Ischaemic	2.1	1.6–5.5	0.003			
Late referral	4.4	2.6–11.1	0.0001	0.4	0.2–1.2	0.2
Gangrene	0.9	0.7–1.4	0.2			
Infection	0.8	0.6–1.2	0.3			
IHD				2.0	1.3–3.3	0.04
ESRD	0.8	0.5–1.7	0.07	0.9	0.8–1.3	0.6

*IHD* ischaemic heart disease; *ESRD* end-stage-renal-disease

## Discussion

Late referral of a DFU to specialized foot services is a common issue worldwide [1]. We have observed after the implementation of the FTP in a specific region in a developed country that the majority of patients were in the ER group (79.5% vs. 20.5%). In addition, the number of LR cases was lower than those reported from previous reports (20.5 vs. 60%) [1]. Even so, patients in the LR group had a greater incidence of type 1 diabetes (19.5 vs. 6.3%), a longer duration of diabetes (approximately 23 vs. 17 years), more cases of ESRD requiring dialysis (26.8 vs. 8.8%), IHD (48.8 vs. 36.5%) and dyslipidemia (92.7 vs. 70.5%) than ER patients.

Nonetheless, LR patient group had a greater rate of severely complicated DFUs (58.5 vs. 20.1%), more episodes associated with ischaemic (75.6 vs. 50.3%), infection (78 vs. 64.8%), and deep to the bone (75.6 vs. 55.3%). Furthermore, the LR group had larger wounds (90 vs. 57.2%) in comparison to ER patients and more cases of gangrene (80.5 vs. 37.7%). It can be concluded that LR patients have a poorer clinical baseline than ER, at presentation to the specialist MDFT. The increased incidence of gangrene, infection, and greater size and deeper wounds in the LR group than in the ER group could in itself be related to the LR to specialised DF service. In addition, infection, size, and depth also negatively influence the chance of healing [7]. Particularly, DFU’s complicated by osteomyelitis often require surgical treatment and long antibiotic therapy [8], and  osteomyelitis is usually associated to non-healing ulcers, and it increased risk of major amputation [9–11].

During the study period, the ER group were observed to go into resolution and heal (89.9 vs. 41.5%), healing times were shorter (approximately 10 vs. 16 weeks), and both rates of minor (17.6 vs. 75.6%) and major (0.6 vs. 36.6%) amputation were lower. Early referral was associated with reduced hospitalization (47.1 vs. 82.9%), and mortality (4.4 vs. 19.5%) than LR. Among the 159 ER presentations, only 1 case resulted in a major amputation, although 50% DFUs were ischaemic, 64% infected and 55% deep to the bone. In addition, ER was an independent predictor of healing, while LR was an independent predictor of minor amputation, major amputation and hospitalization.

Our observations confirm early referral is mandatory for improving healing and limb salvage, to control infection and ischemia. The England and Wales Diabetic Foot Care Audit reported that ER significantly impacts on healing: stating management within 2 weeks from ulcer onset was correlated with a higher rate of healing in comparison to patients who waited more than 2 weeks for their assessment [12]. Smith-Strøm et al. found that DFU patients, retained in the community with a DFU and referred by GPs to DF specialists after 52 days had a reduced rate of healing (> 58%) in comparison to those who had an earlier referral [13]. Faglia et al. reported that LR, combined with ischaemia and infection has a primarily detrimental effect on prognosis: delayed surgical treatment resulted in a higher risk of amputation in comparison with early surgical procedure [14]. These data are in line with our results in which we have tested a new assessment and timing of referral according to DFU’ severity [3]

This study confirms delayed referral, and several variables can predict poorer outcomes of patients with DFUs including diabetes related complications and concomitant co-morbidities. Our data identifies cardiac disease, in this case IHD, as an independent predictor of death, in keeping with the previous literature [15, 16]. Faglia et al. reported IHD was an independent predictor of mortality in a large group of patients with ischaemic DFUs. Another study has shown DFU’s affected by ischaemia, CLI and heart failure have a 30% increase in 1-year mortality rate of approximately 30% in the case of preserved renal function and 55% in the case of ESRD requiring dialysis. Similarly, the presence of ESRD requiring dialysis independently influences the chances of healing as showed in several studies in which dialysis was reported as predictive factor of non-healing and major amputation [17–20]. In addition, the presence of ischaemia was a predictive factor for hospitalization, reflecting the need for peripheral revascularization in patients with ischaemic DFUs.

In summary, there are some key points that can be extrapolated from this current study. The robust implementation of FTP among professionals involved in primary care potentially limits the cases of LR as compared to the published literature. LR is a predictor for poorer outcomes, in its own right and in combination with other factors such as ischaemia and infection. Thus, the timing of referral to specialised DF services defined by the FTP is helpful to achieve positive outcomes for this patient group. The majority of patients who had ER reported less severely complicated DFUs, less cases of amputation, death and hospitalization, and high rate of healing and reduced healing time in comparison to those who had LR.

Therefore, ER should be considered the main goal by the community involved in the DF care. Especially the presence of concomitant co-morbidities (specifically ESRD and cardiac disease) should be considered in the pathway of patients with DFUs due to their impact on ulcer and patients’ outcomes as correctly defined by the FTP.

Data were retrospectively analyzed and based on the results from a single large regional center where a specialized multidisciplinary foot team is operative. Information about the timing of referral and the cases of LR before the implementation of FTP were limited, therefore no comparison is possible. Data on social factors (i.e. living alone, socio-economical status, etc.) which may influence the medical consultation were not available. Even so, we provide an overview on the timing of referral after the implementation of FTP, evaluating its impact of ER and LR on DFU outcomes. Data on autonomic neuropathic complications which may have a determinant role on outcomes were not available. A larger sample could be useful to reinforce these results.

## Conclusion

Late referral is still a current clinical and social barrier in the management of patients with DFUs. Due to the evident advantages of early intervention, there is a need to increase awareness on the management of DFUs among all levels of trained professionals. Our initial experience supports FTP adoption, with comparatively reduced rates of delayed or late referrals to prevent poorer DFU outcomes.

## Data Availability

All data and materials claims and comply with field standards.
